# Wheat Embryo Albumin and Its Peptide Alleviate Acute Exercise Fatigue as Energy Supplement

**DOI:** 10.3390/foods13233866

**Published:** 2024-11-29

**Authors:** Aimei Liao, Xiaoxiao Li, Yanbing Wang, Zhirui Ding, Long Pan, Yinchen Hou, Quanping Liu, Jianzheng Li, Menghui Shang, Jihong Huang

**Affiliations:** 1 Collaborative Innovation Center of Functional Food by Green Manufacturing, School of Food and Pharmacy, Xuchang University, Xuchang 461002, China; aimeiliao@haut.edu.cn; 2 Henan Provincial Engineering Laboratory of Preservation and Breeding of Industrial Microbial Strains, School of Biological Engineering, Henan University of Technology, Zhengzhou 450001, China; 15670630598@163.com (X.L.); 18358960363@163.com (Y.W.); 17839781898@163.com (Z.D.); panlong@haut.edu.cn (L.P.); 15039688081@163.com (M.S.); 3 Food Laboratory of Zhongyuan, Luohe 462300, China; hyc3072@163.com; 4 Zhengzhou Engineering Research Center of Bioactive Peptides, Zhengzhou 450001, China; 13525083037@163.com; 5 School of Food and Bioengineering, Henan University of Animal Husbandry and Economy, Zhengzhou 450046, China; 6 Henan Houyi Industrial Group Co., Ltd, Zhengzhou 451162, China; hyljz999@163.com; 7 State Key Laboratory of Crop Stress Adaptation and Improvement, College of Agriculture, Henan University, Kaifeng 475004, China

**Keywords:** wheat embryo albumin, exercise fatigue, exhaustion swimming, anti-fatigue, energy supplementary material

## Abstract

Wheat embryo albumin (WEA), rich in amino acids with a good balanced proportion, demonstrates plentiful biological activities. The effects of WEA and its peptide with the best antioxidant ability (F3) as a post-workout and pre-workout energy supplement on alleviating acute exercise fatigue were investigated. Under two experimental cases, the exhaustion-to-death swimming time and exhaustion swimming time were determined. Fatigue-related biochemical indexes including lactate dehydrogenase (LDH), the level of blood urea nitrogen (BUN), alanine transaminase (ALT), aspartate transaminase (AST), liver glycogen (LG), and muscle glycogen (MG) were measured with commercial kits. Antioxidant capacity in vivo was analyzed by determining the content of malondialdehyde (MDA), the level of glutathione (GSH), and the activity of superoxide dismutase (SOD) based on colorimetric methods. The results indicated that administration of WEA and F3 post-workout or pre-workout significantly prolonged exhaustive swimming time (*p* < 0.05) and increased the levels of glycogen in the liver and muscle of mice (*p* < 0.05). Meanwhile, WEA and F3 significantly reduced the activities of ALT, AST, and LDH and the level of BUN compared with the ones of mice in an exercise fatigue model (*p* < 0.05). Additionally, in comparison with the model group, supplements of WEA and F3 obviously decreased the content of MDA while enhancing the activity of SOD and the level of GSH both in the liver and muscle of mice. These results demonstrated that WEA and F3 can mitigate exercise fatigue and are conducive to recovery from fatigue in exhausted mice. It suggests that WEA and its peptide F3 could be a promising energy supplementary material against fatigue caused by continuous or high-intensity exercise.

## 1. Introduction

More and more people are suffering from a state of sub-health due to multiple factors such as a modern fast-paced life style, irregular work and rest, inadequate physical activity, unreasonable exercise, insufficient sleep, an unhealthy diet, high psychological pressure, mental tension, and so on [1]. As one of the prominent features of sub-health, fatigue is generally defined as a disabling symptom characterized by a persistent feeling of tiredness, weakness, or lack of energy and difficulty in maintaining voluntary activity [2]. Fatigue is usually caused by physiological or pathological actions such as intense physical activity, long-time continual exercise, and prolonged hard mental work, as well as medical conditions [3]. Correspondingly, fatigue usually results in physical dysfunction, sleep weakness, energy imbalance, aging, anxiety, depression, and cognitive impairment, as well as various diseases including cancer and multiple sclerosis [4,5,6]. General fatigue can also be classified as peripheral or central fatigue, which occur within the skeletal muscle and central nervous system (CNS), respectively. Exercise fatigue as a typical non-pathological physiological phenomenon is related to both peripheral muscle and the state of the CNS. Appropriate exercise helps to adapt and improve muscle strength, increase metabolism, promote better energy utilization and fat burning, improve cardiovascular health, and so on. Short-lived fatigue is considered as a self-protective mechanism to avoid a large depletion of stored energy and the mechanical injury of muscle [7]. However, prolonged fatigue induced by intense or continuous strenuous physical exercise results in muscle pain and tiredness and leads to weak physical capability, breaking the balance between strength and energy output, decreasing endurance after continuous or strenuous exercise, and even to an inability to perform usual activities or Karoshi (death resulted from overwork) [8,9,10]. Therefore, effective anti-fatigue treatment strategies are urgently required.

Multiple kinds of medications have been applied to enhance exercise performance or relieve fatigue after intense exercise and improve organic recovery. Caffeine, a type of naturally occurring stimulant, is consumed through beverages including coffee, tea, and energy drinks to temporarily ward off sleepiness and restore alertness, enhancing physical performance due to its ability to increase endurance and reaction times and alleviate perceived exertion [11]. Vitamins (Vitamin E and Vitamin C) [12] and crucial types of Omega-3 fatty acids such as Eicosapentaenoic Acid (EPA) and Docosahexaenoic Acid (DHA) have been demonstrated to obviously reduce exercise fatigue owing to their antioxidant and anti-inflammatory activities [13]. Many traditional Chinese medicines, for instance Astragali, Ginseng, and Codonopsis, have been indicated to treat fatigue via pathways related to antioxidant ability, anti-inflammatory activity, and the regulation of metabolites and gut microbiota, which were demonstrated to be related to anti-hyperlipidemia therapies [14,15,16,17]. Nevertheless, different disadvantages of the medications mentioned above, such as low absorption, limited bioavailability and effects, and toxicity or some side effects, have restricted their widespread application [18,19]. Nowadays, anti-fatigue natural active ingredients such as polysaccharides, protein, and polypeptides have become more attractive due to their obvious efficacy, high safety, and few side effects [20,21,22,23].

Wheat germ, a kind of by-product of wheat flour processing, is known as the natural treasure of nutrition because it is rich in nutritional active ingredients such as unsaturated fatty acid, glutathione, polysaccharide, and protein. Wheat germ protein is mainly composed of wheat germ globulin (WGG) and wheat germ albumin (WGA). WGG has been proved to be beneficial for health, including antioxidant and anti-aging effects, immunoregulation, cognitive impairment protection, the prevention of alcohol-induced liver injury, and so on [24,25,26,27]. However, the nutritional healthy functions of WEA, especially as a kind of energy supplement, have been rarely investigated. 

WEA and its peptide were previously prepared, and their antioxidant activities were measured [28]. In the present study, the effects of WEA and its peptide as a pre-workout and post-workout energy supplement on delaying acute fatigue induced by high-intensity exercise were investigated.

## 2. Materials and Methods

### 2.1. Chemicals and Reagents

Panax ginseng was provided from Anhui Yuzhiyuan Chinese Medicine Drinking Tablets Co., Ltd. (Bozhou, China). Dinitrobenzoic acid (DTNB), ethylenediaminetetraacetic acid (EDTA), and sodium lauryl sulfate (SDS) were purchased from Beijing Solarbio Science & Technology Co., Ltd. (Beijing, China). Tetraethoxypropane, nitroblue tetrazolium (NBT), and methionine (MET) were obtained from Shanghai Macklin Biochemical Technology Co., Ltd. (Shanghai, China). Sulfosalicylic acid was supplied by Shanghai Yuanye Biotechnology Co., Ltd. (Shanghai, China). Acetic acid, Sodium acetate, Trichloroacetic acid, NaOH, Ammonium sulfate, Ethanol absolute, and Riboflavine were bought from Tianjing Kemiou Chemical Reagent Co.Ltd (Tian Jing, China). Tert-Butylalcohol (TBA) was commercially obtained from Sinopharm Chemical Reagent Co., Ltd. (Shanghai, China). Reduced GSH was purchased from Shandong Xiya Chemical Co., Ltd. (Linying, China). Kits for the measurement of liver glycogen, muscle glycogen, blood urea nitrogen (BUN), lactate dehydrogenase (LDH), alanine aminotransferase (ALT), and aspartate aminotransferase (AST) were obtained from the Nanjing Jiancheng Bioengineering Institute (Nanjing, China). All other reagents not mentioned here were analytical grade.

### 2.2. Preparation of WEA and Peptide F3

WEA and F3 were prepared as previously recorded by Liao et al. [29]. Briefly, after thorough degreasing, a specific quantity of wheat germ was dissolved in deionized water with a weight-to-volume ratio of 1:10. This mixture was subjected to agitation at a temperature of 30 °C for a duration of 1.5 h. Post agitation, the solution was transferred to the centrifuge (3k-18, Sigma, Berlin, Germany) and centrifuged at 4 °C for a period of 20 min at a rotational speed of 8000 revolutions per minute (rpm). Following centrifugation, the supernatant was carefully harvested and subsequently freeze-dried to acquire the final WEA by a freezing drier (Scientz-10N, Ninbo Scientz Biotechnology Co., Ltd, Ninbo, China). The WEA (2% of substrate concentration) was enzymatic hydrolyzed by papain with 8000 U/g at 55 °C and pH 6, to prepare WEA hydrolysates that were separated by ultrafiltration to obtained the peptide F3 with a molecular weight of 3–5 kDa. After lyophilization, the component F3 was stored at −80 °C for further analysis. 

### 2.3. Animals and Treatments

Male Kunming mice (6~8-week-old, body weight 20 ± 2 g) were purchased from Huaxing Laboratory Animal Farm (Zhengzhou, China). The mice were maintained at a relative humidity of 50 ± 10% and a relative temperature of 25 ± 2 °C. Well-ventilated with 12:12 h of alternating light and dark, the mice were freely provided with a chow diet and aseptic tap water. All animal experiments were conducted following the guidelines set by the institutional animal care and use committee of Henan University of Technology (license number: SCXK (Yu) 20190002).

**Figure 1:**
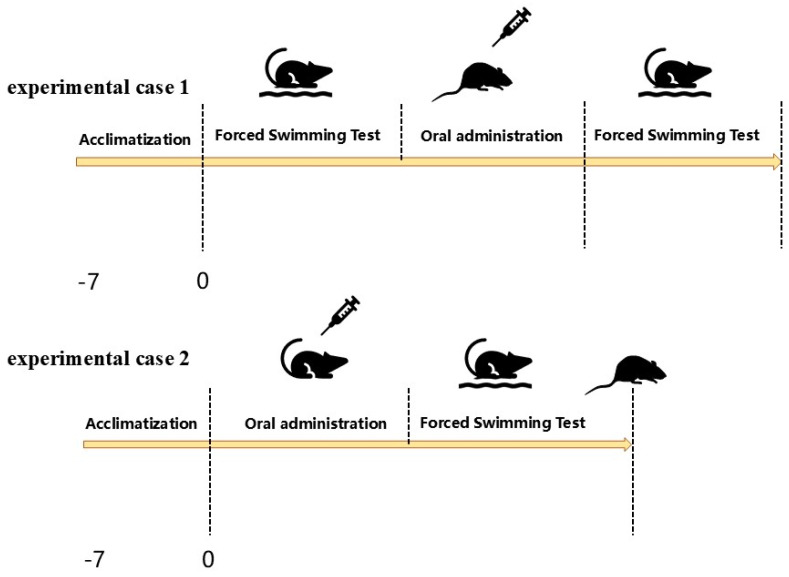
The experiment design of exhaustive swimming and the energy supplement.

After seven days of adjustable feeding, the mice were randomly divided into two experimental cases (*n* = 72), experimental case 1 and experimental case 2, in which WEA and F3 were applied as post-workout and pre-workout energy supplements (Figure 1). Then, the mice of experimental case 1 were randomly divided into four groups (*n* = 18): Normal control (CG) group, WEA-treated (WEA) group: mice were administrated with WEA (50 mg/kg body weight) by oral gavage; F3-treated (F3) group: mice were given peptide F3 (50 mg/kg body weight) by oral gavage; and positive control (PG) group: mice were orally administrated with 50 mg American ginseng/kg body weight by gavage [30,31]. Experimental case 2 were also grouped as described above. The mice in the CG groups were given saline solution with the same volume.

### 2.4. Exhaustion-to-Death Swimming Experiment

Eight mice were selected from each of the four groups in experimental case 1 at random. These mice swam freely in the swimming tank (60 × 60 × 40 cm) without weights, and they were considered to have reached a state of exhaustion if they failed to surface to breathe through their nose for over 10 s. They were quickly picked up. Next, these mice post-workout were given a gavage, and half an hour after the gavage they were again placed in the swimming tank for non-weight-bearing swimming until they died of over-exertional fatigue. The time of the two swims before and after were recorded separately. Eight mice were randomly selected from each of the four groups in experimental case 2 and given different gavage treatments according to the groups. After a 30 min pre-workout gavage, the mice were deposited in the swimming tank for non-weight-bearing swimming until they died due to exercise fatigue, and the swimming time was recorded.

### 2.5. Exhaustion Swimming Experiment

The remaining 10 mice in the four groups of experimental case 1 were placed in the same swimming tank for non-weight-bearing swimming. When the mice did not surface for 10 s, they were fished out and given a gavage treatment, and after 30 min of gavage, they were allowed to swim again. When the mice again did not surface for 10 s, they were quickly fished out, and the time of the two swims before and after was recorded separately. The remaining 10 mice in the four groups of experiment case 2 were placed in the swimming tank for non-weight-bearing swimming 30 min after being given different gavage treatments according to the groups, and when the mice did not surface for 10 s, they were fished out, and the time was recorded (Figure 1).

### 2.6. Blood and Sample Collection

After 0.5 h of the exhaustive swimming experiment, the eyeballs were then removed to obtain whole blood under formaldehyde. The obtained blood was kept at room temperature for two hours before centrifuging it at 4 °C, 3000 rpm/min for 5 min with a 3k-18 cryogenic high-speed centrifuge (Sigma, Berlin, Germany). Then the serum was taken and reposited at −80 °C for further analysis. Organ samples such as liver, heart, spleen, lung, kidney, muscle, and cecum were removed by rapid dissection, washed with sterilized pre-cooled saline, weighed, divided, and stored in a −80 °C refrigerator.

### 2.7. Determination of Fatigue-Related Biochemical Indexes

Biochemical indicators of fatigue such as the content of blood urea nitrogen (BUN), lactate dehydrogenase (LDH), aspartate transaminase (AST), alanine transaminase (ALT), liver glycogen (LG), and muscle glycogen (MG) were determined according to the manufacturer’s instructions of the detection kit.

### 2.8. Determination of Antioxidant-Related Biochemical Indexes

Liver/muscle tissue and pre-cooled saline were added at 1:9 (*v*:*v*). The tissues were cut with scissors and ground well with a homogenizer, all operations being performed on ice. The 3k-18 cryogenic high-speed centrifuge (Sigma, Berlin, Germany) parameters were set to 4 °C and 8000 rpm/min. Tissue homogenates were separated by centrifuging at 4 °C, 3000 rpm/min for 10 min, and the supernatant was taken for subsequent measurement of the activity of SOD, the content of GSH, and MDA. SOD activity was measured as recorded by Stewert et al. [32]. The content of GSH was determined as recorded by Nakajima et al. [33]. The level of MDA was measured by the TBA method [32].

### 2.9. Statistical Analysis

Data processing and graph creation were conducted with Origin 2018 (Northampton, MA, USA), and data were expressed as means values ± standard deviation (SD). A one-way analysis of variance (ANOVA) was applied to compare the means between samples, and Tukey’s test was employed for multiple comparisons to assess the statistical significance of the differences. Statistically significant differences (*p* < 0.05) were labeled as different letters.

## 3. Results and Discussion

### 3.1. Effects of WEA and F3 on Exhaustion-to-Death Swimming Times of Mice

Generally, exhaustive swimming experiments can assess the level of fatigue in exercise, while objectively reflecting the body’s physical capabilities. In addition, this experiment has been widely carried out in animal models to reflect anti-fatigue effects [34]. The experimentally longer swim times observed reflect improved endurance and a reduced susceptibility to fatigue [35,36].

**Figure 2:**
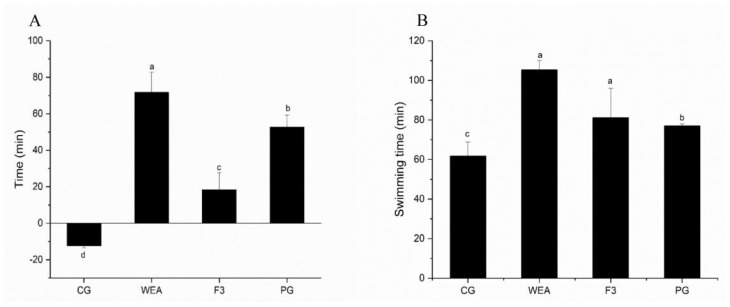
Effects of supplements of WEA and F3 by oral gavage on exhaustion-to-death swimming time. (**A**) Increment in swimming time of mice in experimental case 1 after oral administration. (**B**) Swimming time of mice in experimental case 2. Means with different letters mean significant difference (*n* = 8, *p* < 0.05).

The effects of supplements of the prepared WEA and its peptide F3, with the best antioxidant capacity, by oral gavage on exhaustion-to-death swimming time are summarized in Figure 2. In experimental case 1, compared to the swimming time before oral supplement by gavage, the swimming time of mice after gavage in the CG group was shortened by 12.3 min, while the swimming time of mice in the WEA, F3, and PG group was prolonged by 71.7 min, 18.3 min, and 52.7 min, respectively. In comparison to the CG group, the swimming time of mice in the WEA, F3, and PG groups was observably prolonged (*p* < 0.05). Meanwhile, the swimming time of mice in the WEA group was significantly longer than the one of mice in the F3 group (*p* < 0.05) (Figure 2A).

In experimental case 2, compared to the swimming time of mice in the CG group, the swimming time was extended by 41.29%, 31.27%, and 21.36% by the oral administration of WEA, F3, and PG as a pre-workout energy supplement, respectively (*p* < 0.05). Additionally, the swimming time of mice in the WEA group was markedly longer than that in the F3 group (*p* < 0.05) (Figure 2B). It indicated that both WEA and F3 could prolong the time of swimming and improve the swimming endurance of mice, but the effect of WEA was better.

### 3.2. Effects of WEA and F3 on Exhaustion-Swimming Times of Mice

**Figure 3:**
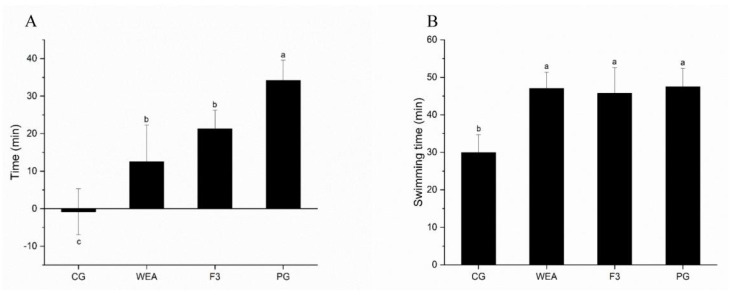
Effects of post-workout or pre-workout supplement of WEA and F3 on exhaustion-swimming times of mice. (**A**) Increment in swimming time of mice in experimental case 1 after oral administration of supplement. (**B**) Swimming time of mice in experimental case 2. Means with different letters mean significant difference (*n* = 8, *p* < 0.05).

The beneficial effects of post-workout or pre-workout supplements of WEA and F3 are as illustrated in Figure 3. In experimental case 1, compared with the swimming time before oral gavage, it was visibly shortened after gavage in the CG group; the swimming time of mice was significantly prolonged by 12.5 min, 21.3 min, and 34.2 min by oral administration of WEA, F3, and American ginseng in the WEA, F3, and PG groups, respectively. The swimming time of mice in the WEA, F3, and PG groups was distinctly extended in comparison to that of the CG group (*p* < 0.05), but there was no significant difference between the administered groups with WEA and its peptide F3 (p>0.05) (Figure 3A).

In experimental case 2, the swimming time of mice in the WEA, F3, and PG groups was prolonged by 36.66%, 34.49%, and 36.88%, respectively (*p* < 0.05) compared to the CG group. However, there was not a statistical difference among the three groups that were administered WEA, F3, and American ginseng as a pre-workout energy supplement (*p* > 0.05). It indicates that oral gavage of both WEA and F3 displayed similar energy and nutritional supplementary effects to American ginseng against exercise fatigue (Figure 3B).

### 3.3. Effects of WEA and F3 on Liver and Muscle Glycogen Contents of Mice

Glycogen depletion is a primary cause of fatigue during exercise [37]. Glycogen is usually classified into hepatic glycogen and muscular glycogen; the former holds blood glucose levels, and the latter offers energy for muscle contraction [38]. ATP is the body’s most direct energy source during exercise, and the main substances that produce ATP are sugars, fats, and proteins. After intensive exercise, there is a simultaneous increase in the metabolic activity of energy substances in the body, and the higher content of muscle glycogen is first used for oxidative energy supply. At this time, the proportion of blood glucose for energy supply increases gradually with the prolongation of exercise time, and liver glycogen begins to break down with the decrease of blood glucose concentration in the body, thus maintaining the supply of blood glucose to the brain and the body’s energy [39].

**Figure 4:**
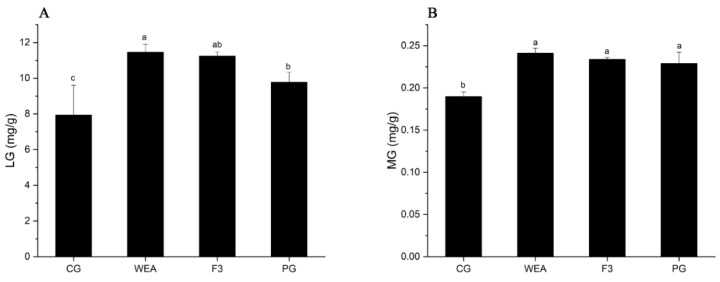
The glycogen content of mice in experimental case 1. (**A**) Liver glycogen, LG, (**B**) muscle glycogen, MG. Means with different letters mean obvious difference (*n* = 8, *p* < 0.05).

In experimental case 1, compared with the CG group, the liver glycogen content of mice in the WEA, F3, and PG groups was significantly (*p* < 0.05) increased by 30.71%, 29.42%, and 18.83%, respectively, and the liver glycogen content of mice in the WEA group was obviously higher than that in the PG group (*p* < 0.05). In addition, the muscle glycogen content of mice in the WEA, F3, and PG groups was markedly (*p* < 0.05) increased by 21.35%, 18.89%, and 17.09%, respectively, compared to the CG group, but not differentiated among the three administered groups (*p* > 0.05) (Figure 4).

**Figure 5:**
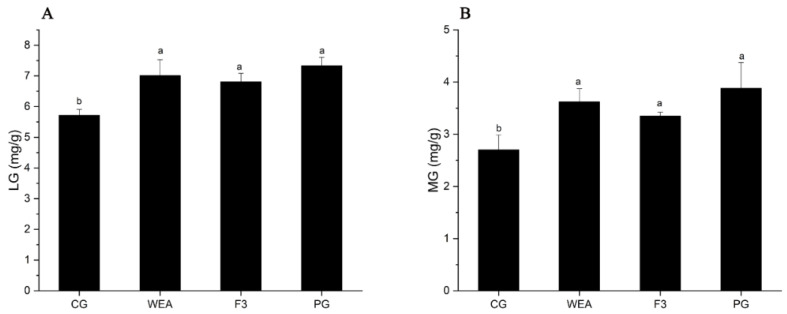
The glycogen level of mice in experimental case 2. (**A**) Liver glycogen, LG, (**B**) muscle glycogen, MG. Means with different letters represent significantly different (*n* = 10, *p* < 0.05).

In experimental case 2, in comparison to the CG group, the hepatic glycogen level of mice in the WEA, F3, and PG groups was visibly increased (*p* < 0.05) by 18.43%, 16.05%, and 21.99%, respectively. And there was no significant difference among the three administered groups (*p* > 0.05). Furthermore, compared to the CG group, the muscle glycogen content of mice in the WEA, F3, and PG groups was significantly (*p* < 0.05) enhanced by 25.42%, 19.31%, and 30.39%, respectively, but there was no obvious difference among the three treated groups (*p* > 0.05). These results indicated that WEA and F3 significantly improved the levels of hepatic glycogen and muscle glycogen in mice, and the WEA was more effective than F3, but not differentially (Figure 5). 

Although the supplementation of WEA or F3, which consist of amino acid, did not directly provide glucose for the biosynthesis of glycogen in liver or muscle, the administration of protein or peptides might influence the efficiency of glycogen consumption and replenishment. Researchers have indicated that the recruitment of glucose or whey protein hydrolysates obviously decreases the consumption of muscle glycogen. These studies suggested that hydrolysates of whey protein could stimulate insulin release, which in turn motivates glycogen synthase and pathways in skeletal muscle such as Akt/PKB, PKC ζ [40,41]. In the present study, WEA or F3 possibly activates the release of insulin to upgrade the biosynthesis of glycogen, which may be the potential mechanism of anti-fatigue activities of WEA or F3.

### 3.4. Effects of WEA and F3 on the Liver Function of Mice

Severe physical exercise has been reported to cause the rapid atrophy of liver cells, which can lead to liver dysfunction [42]. Excessive alanine transaminase (ALT) and aspartate transaminase (AST) are essential indexes of cell damage of the liver, and thus they reflect the characteristic response during severe exercise [43].

**Figure 6:**
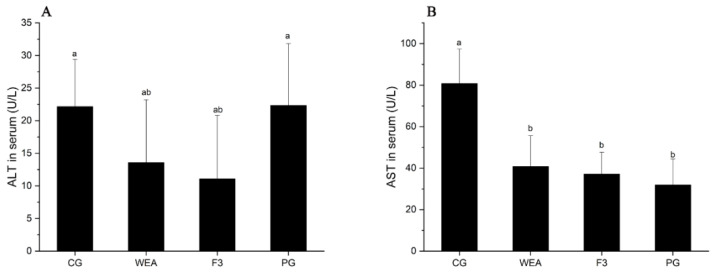
The ALT and AST activities in serum of mice in experimental case 1. (**A**) Alanine transaminase, ALT, (**B**) aspartate transaminase, AST. Means with different letters mean significant difference (*n* = 8, *p* < 0.05).

In experimental case 1, the activity of ALT in mice administrated with WEA and F3 was significantly reduced (*p* < 0.05) by 38.73% and 49.98%, respectively, compared to the CG group. There was no significant difference between the activity of ALT of mice in the PG group and that of the CG group (*p* > 0.05). In addition, the activity of AST in mice in the WEA, F3, and PG groups was distinctly (*p* < 0.05) reduced by 49.55%, 54.02%, and 60.54%, respectively, compared with the CG group. However, there was no difference between the three administered groups (*p* > 0.05) (Figure 6).

**Figure 7:**
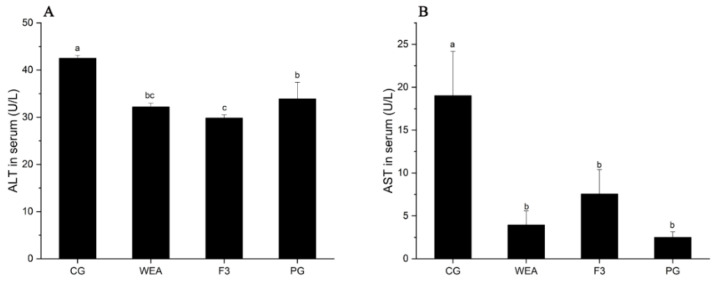
The ALT and AST activities in the serum of mice in experimental case 2. (**A**) Alanine transaminase, ALT, (**B**) aspartate transaminase, AST. Means followed by different letters are significantly different (*n* = 10, *p* < 0.05).

In experimental case 2, compared with the CG group, the ALT activity of mice in the WEA, F3, and PG groups was significantly lower (*p* < 0.05) by 24.31%, 29.83%, and 20.25%, respectively, and the ALT activity of mice in the PG group was significantly increased compared to that in the F3 group (*p* < 0.05), but the ALT activity of mice in the WEA and F3 groups did not indicate significant differences (p>0.05). Additionally, the AST content of mice in the WEA, F3, and PG groups was significantly reduced (*p* < 0.05) by 79.26%, 60.28%, and 86.83%, respectively, compared with that in the CG group, and the ALT content of mice in the PG and WEA groups was lower than that in the F3 group (*p* > 0.05) (Figure 7). Therefore, the results of this work suggest that WEA and F3 may exert anti-fatigue effects by protecting cells against excessive exercise damage and enhancing the body’s endurance.

### 3.5. Effect of WEA and F3 on LDH and BUN Levels of Mice

Lactate dehydrogenase (LDH) and blood urea nitrogen (BUN) are representative biochemical indicators of fatigue and are more commonly used in anti-fatigue studies [44,45]. LDH is normally found in muscle cells and is critical in an organism’s energy metabolism. When the LDH changes, the energy metabolism of the organism is directly affected. When homeostasis within the body is greatly altered, LDH is released from the muscle cells into the bloodstream, which causes a change in pH, leading to muscle damage [46,47]. BUN is mainly produced through the metabolism of protein and amino acid metabolism [48]. It has also been found that when the body’s exercise reaches a certain intensity, energy produced by sugar and fat is insufficient to meet the body’s demands. As a result, proteins and amino acids become alternative sources of catabolism, leading to increased levels of BUN [48,49,50].

**Figure 8:**
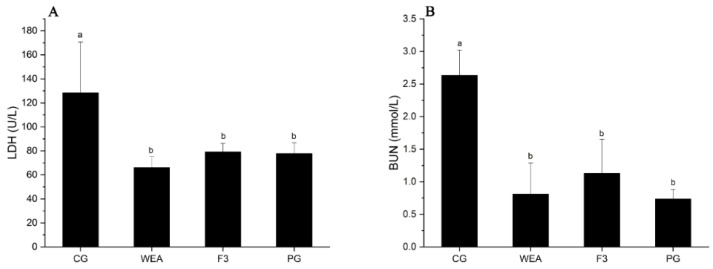
LDH and BUN activities in serum of mice in experimental case 1. (**A**) Lactate dehydrogenase, LDH, (**B**) blood urea nitrogen, BUN. Means with different letters are significantly different (*n* = 8, *p* < 0.05).

In experimental case 1, the LDH content of mice administrated with WEA, F3, and American ginseng was significantly (*p* < 0.05) reduced (48.58%, 38.43%, and 39.47%, respectively), in comparison to the mice offered saline solution, but there was no differentiation among the three administered groups (*p* > 0.05). Additionally, the BUN content of mice in the WEA, F3, and PG groups was distinctly (*p* < 0.05) reduced by 69.25%, 57.07, and 72.07%, respectively, when compared to the CG group, but there was no differentiation among the three administration groups (*p* > 0.05) (Figure 8).

**Figure 9:**
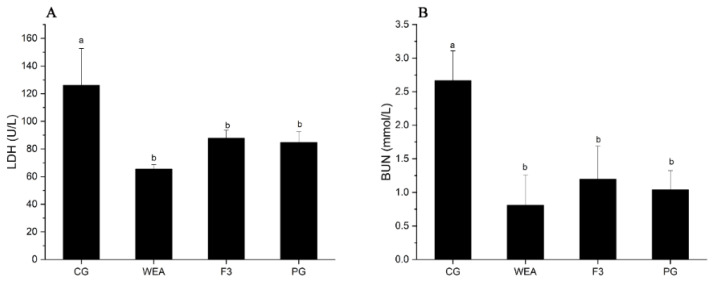
The LDH and BUN activities in the serum of mice in experimental case 2. (**A**) Lactate dehydrogenase, LDH, (**B**) blood urea nitrogen, BUN. Means followed by different letters are significantly different (*n* = 10, *p* < 0.05).

In experimental case 2, the LDH content of mice in the WEA, F3, and PG groups was obviously (*p* < 0.05) reduced by 48.03%, 30.38%, and 32.83%, respectively, compared to the CG group. However, there was no difference between the three administration groups (*p* > 0.05). In addition, the BUN content of mice in the WEA, F3, and PG groups was significantly (*p* < 0.05) reduced by 69.58%, 55%, and 60.94%, respectively, compared with that of the CG group, but there was no differentiation among the three administered groups (*p* > 0.05) (Figure 9). It can be seen that WEA reduces the accumulation of LDH and BUN, suggesting that administration with WEA and F3 reduces fatigue-induced muscle damage and cellular damage. The effect of WEA is better than that of F3, but the two are not differentiated.

### 3.6. Effect of WEA and F3 on Oxidative Stress Parameters of Mice

Previous studies have shown that oxidative stress is associated with chronic fatigue and fatigue-related diseases [51]. Excessive exercise produces excessive reactive oxygen species, leading to impaired muscle contraction and tissue damage associated with oxidative stress [52]. SOD and GPX are major components of the enzymatic antioxidant defense system that attenuates the damage caused by excessive oxidative stress by reducing free radicals [49,53]. MDA is an indicator of lipid peroxidation, which is a degradation product of polyunsaturated fatty acids in the presence of free radicals in the membrane [54]. Therefore, the degree of cellular oxidative stress can be reflected by the level of MDA in vivo. In this study, the antioxidant capacity of WEA and F3 was evaluated by measuring SOD activity and GSH and MDA levels.

In experimental case 1, SOD activity was significantly elevated (*p* < 0.05) in the livers of mice in the WEA, F3, and PG groups compared to the CG group (48.84%, 45.02%, and 48.84%, respectively), but there was no differentiation among the three administered groups (*p* > 0.05). GSH levels were significantly (*p* < 0.05) elevated (61.68%, 63.72%, and 63.39%) in mice of the WEA, F3, and PG groups, respectively, compared to the CG group, but not differentially (*p* > 0.05) among the three administered groups. In addition, in comparison to the mice in the CG group, the MDA content of mice in the WEA, F3, and PG groups was distinctly decreased (*p* < 0.05) by 82.39%, 61.93%, and 53.41%, respectively, but there was no differentiation among the three administered groups (*p* > 0.05). SOD activity in the muscles of mice in the WEA, F3, and PG groups was obviously (*p* < 0.05) enhanced by 63.77%, 81.2%, and 78.63, respectively, compared to the CG group. GSH levels in mice in the WEA, F3, and PG groups were significantly (*p* < 0.05) increased by 43.79%, 58%, and 58.98%, respectively, compared to the CG group. In addition, the MDA levels of mice in the WEA, F3, and PG groups were significantly reduced (*p* < 0.05) by 64.18%, 77.98%, and 78.17%, respectively, compared with the CG group (Table 1).

In experimental case 2, SOD activity was significantly (*p* < 0.05) elevated (35.37%, 27.42%, and 38.96%) in the livers of mice in the WEA, F3, and PG groups, respectively, compared to the CG group, but was not differentiated between the three administered groups (p>0.05). GSH content was significantly elevated (*p* < 0.05) in mice of the WEA, F3, and PG groups compared to the CG group (54.33%, 36.23%, and 75.82%, respectively). In addition, the MDA content of mice in the WEA, F3, and PG groups was significantly reduced (*p* < 0.05) by 68.10%, 60.74%, and 77.61%, respectively, compared to the CG group, but not differentially among the three administered groups (*p* > 0.05). SOD activity in the muscles of mice in the WEA, F3, and PG groups was significantly (*p* < 0.05) elevated by 75%, 78.03%, and 75.97%, respectively, compared to the CG group. GSH content was significantly elevated (*p* < 0.05) in mice of the WEA, F3, and PG groups compared to the CG group (66.09%, 41.33%, and 60.63%, respectively). In addition, the MDA content of mice in the WEA, F3, and PG groups was significantly (*p* < 0.05) lower (73.57%, 55.71%, and 75%) compared to the CG group. The experimental results showed that WEA and F3 reduced fatigue-induced oxidative stress (Table 2 and Table 3).

## 4. Conclusions

This study evaluated the anti-acute exercise fatigue effects of WEA and F3 as post-workout and pre-workout energy supplements through establishing a mouse model of acute exercise fatigue. The results revealed that under two conditions, WEA and F3 indicated anti-fatigue activities, and WEA was more effective at improving the swimming endurance of mice than F3, replenishing liver glycogen and muscle glycogen reserves and slowing down the accumulation of LDH and BUN content in serum in a timely manner in the bodies of mice.

Moreover, WEA and F3 potentially mitigated acute exercise fatigue by protecting cells from excessive exercise damage and enhancing body endurance. By the measurement of antioxidant indexes in the liver and skeletal muscle, WEA and F3 were found to prevent lipid oxidation, enhance antioxidant enzyme activities, and enable the body better to maintain the balance between oxidative and antioxidant systems.

Thus, it is suggested that both WEA and F3 may be developed as innovative sports energy supplement products in the fight against acute exercise fatigue. Therefore, this study provides different perspectives for the development of relevant functional foods in the field of the pharmaceutical and food industries. However, the exact and detailed mechanism of action of WEA and F3 in combating acute exercise fatigue, including anti-fatigue, by regulating the gut flora and metabolites needs to be further explored, which is still going on in our lab.

## Figures and Tables

**Table 1:** The SOD activity and GSH and MDA content in the liver and skeletal muscle of mice in experimental case 1.

	SOD (U/mg pro)	GSH (μmol/g pro)	MDA (nmol/g pro)
Liver			
CG	2.87 ± 0.36 ^b^	0.41 ± 0.25 ^b^	1.76 ± 0.48 ^a^
WEA	5.61 ± 0.50 ^a^	1.07 ± 0.20 ^a^	0.31 ± 0.11 ^b^
F3	5.22 ± 0.28 ^a^	1.13 ± 0.32 ^a^	0.67 ± 0.23 ^b^
PG	5.61 ± 0.15 ^a^	1.12 ± 0.41 ^a^	0.82 ± 0.53 ^b^
Skeletal muscle			
CG	0.25 ± 0.06 ^c^	8.15 ± 0.56 ^c^	5.36 ± 1.13 ^a^
WEA	0.69 ± 0.13 ^b^	14.50 ± 0.95 ^b^	1.92 ± 0.45 ^b^
F3	1.33 ± 0.42 ^a^	19.40 ± 0.23 ^a^	1.18 ± 0.23 ^b^
PG	1.17 ± 0.13 ^a^	19.87 ± 0.53 ^a^	1.17 ± 0.20 ^b^

Values are expressed as mean ± SD. Values with different superscript letters (^a^, ^b^, ^c^) in the same column represent a significant different (*p* < 0.05).

**Table 2:** The SOD activity and GSH and MDA content in the liver of mice in experimental case 2.

	SOD (U/mg pro)	GSH (μmol/g pro)	MDA (nmol/g pro)
CG	8.68 ± 0.18 ^c^	1.32 ± 0.22 ^c^	3.26 ± 0.68 ^a^
WEA	13.43 ± 0.17 ^a^	2.89 ± 0.79 ^b^	1.04 ± 0.79 ^b^
F3	11.96 ± 0.47 ^b^	2.07 ± 0.23 ^bc^	1.28 ± 0.54 ^b^
PG	14.22 ± 0.57 ^a^	5.46 ± 0.60 ^a^	0.73 ± 0.38 ^b^

Values are expressed as mean ± SD. Values with different superscript letters (^a^, ^b^, ^c^) in the same column represent a significant different (*p* < 0.05).

**Table 3:** The SOD activity and GSH and MDA content in the skeletal muscle of mice in experimental case 2.

	SOD (U/mg pro)	GSH (μmol/g pro)	MDA (nmol/g pro)
CG	0.68 ± 0.32 ^c^	3.52 ± 0.24 ^c^	1.40 ± 0.08 ^a^
WEA	2.72 ± 0.23 ^a^	10.38 ± 1.90 ^a^	0.37 ± 0.20 ^b^
F3	1.73 ± 0.14 ^b^	6.00 ± 0.73 ^b^	0.62 ± 0.23 ^b^
PG	2.83 ± 0.09 ^a^	8.94 ± 1.83 ^a^	0.35 ± 0.05 ^b^

Values are expressed as mean ± SD. Values with different superscript letters (^a^, ^b^, ^c^) in the same column represent a significant different (*p* < 0.05).

## Data Availability

The original contributions presented in the study are included in the article, further inquiries can be directed to the corresponding author.
